# Molecular detection and phylogenetic analysis of lumpy skin disease virus from outbreaks in Uganda 2017–2018

**DOI:** 10.1186/s12917-020-02288-5

**Published:** 2020-02-21

**Authors:** Sylvester Ochwo, Kimberly VanderWaal, Christian Ndekezi, Joseph Nkamwesiga, Anna Munsey, Sarah Gift Witto, Noelina Nantima, Franklin Mayanja, Anna Rose Ademun Okurut, David Kalenzi Atuhaire, Frank Norbert Mwiine

**Affiliations:** 1 College of Veterinary Medicine, Animal resources and Biosecurity, Makerere University, P.O.BOX 7062 Kampala, Uganda; 2 College of Veterinary Medicine, University of Minnesota, 1365 Gortner Avenue St. Paul, MN, Minneapolis, MN 55108 USA; 3 Ministry of Agriculture Animal Industry & Fisheries, Berkley Ln, Entebbe, Uganda

**Keywords:** Molecular detection, GPCR, Phylogenetic analysis, Lumpy skin disease, Uganda

## Abstract

**Background:**

Lumpy skin disease (LSD) is an infectious viral disease of cattle caused by a *Capripoxvirus*. LSD has substantial economic implications, with infection resulting in permanent damage to the skin of affected animals which lowers their commercial value. In Uganda, LSD is endemic and cases of the disease are frequently reported to government authorities. This study was undertaken to molecularly characterize lumpy skin disease virus (LSDV) strains that have been circulating in Uganda between 2017 and 2018. Secondly, the study aimed to determine the phylogenetic relatedness of Ugandan LSDV sequences with published sequences, available in GenBank.

**Results:**

A total of 7 blood samples and 16 skin nodule biopsies were screened for LSDV using PCR to confirm presence of LSDV nucleic acids. PCR positive samples were then characterised by amplifying the GPCR gene. These amplified genes were sequenced and phylogenetic trees were constructed. Out of the 23 samples analysed, 15 were positive for LSDV by PCR (65.2%). The LSDV GPCR sequences analysed contained the unique signatures of LSDV (A11, T12, T34, S99, and P199) which further confirmed their identity. Sequence comparison with vaccine strains revealed a 12 bp deletion unique to Ugandan outbreak strains. Phylogenetic analysis indicated that the LSDV sequences from this study clustered closely with sequences from neighboring East African countries and with LSDV strains from recent outbreaks in Europe. It was noted that the sequence diversity amongst LSDV strains from Africa was higher than diversity from Eurasia.

**Conclusion:**

The LSDV strains circulating in Uganda were closely related with sequences from neighboring African countries and from Eurasia. Comparison of the GPCR gene showed that outbreak strains differed from vaccine strains. This information is necessary to understand LSDV molecular epidemiology and to contribute knowledge towards the development of control strategies by the Government of Uganda.

## Background

Lumpy skin disease virus (LSDV) belongs to the genus *Capripoxvirus*, subfamily *Chordopoxvirinae* and family *Poxviridae* [1]. Lumpy skin disease (LSD) is characterised by enlarged superficial lymph nodes, fever and growth of firm skin nodules that become open wounds leading to secondary bacterial infections, sometimes resulting in death of clinically ill cattle [2, 3]. Internationally, LSD leads to financial losses due to trade restrictions applied to live cattle, sheep, goats and animal products from affected countries [4]. In Eastern Africa, economic loss is due to restrictions in animal movement, vaccination costs, and costs of treating secondary bacterial infections. The direct economic loss due to LSD is estimated at 141 USD per lactating head of cattle while the vaccination cost is 5 USD per cow [5].

In different epizootic circumstances, LSD morbidity and mortality fluctuate between 3 to 85%, and between 1 and 40% respectively, based on whether the outbreak is in an endemic or non-endemic region [6, 7]. These broad ranges of morbidity and mortality are likely owing to genetic differences in livestock breeds resulting in varying susceptibility to disease, variable virulence of viral isolates and varying effectiveness of transmission of insect vectors involved in LSDV transmission [2, 4, 8].

In 1929, LSD was first recorded in sub-Saharan Africa [9], spreading to most areas of Africa by the late 1980s [10, 11]. The disease then spread to Middle East nations and more recently spread to Southeast Europe from the Middle East [12], affecting member states of the European Union [13, 14] and several other Balkan countries [15, 16]. In Uganda, LSD is considered endemic and numerous outbreaks occur each year and some of these outbreaks may not be reported to government veterinary authorities. LSD in Uganda is controlled through quarantine restrictions and vaccination with live attenuated vaccines. Vaccination against LSD in Uganda is a responsibility of the livestock farmers rather than government. This may lead to misuse or abuse of vaccines bringing about co-infection and recombination of vaccine strains with virulent strains [17], resulting in virulent reversal of vaccine strains, which may lead to more outbreaks.

Effective control or eradication of LSD in endemic and non-endemic regions needs fast and precise diagnostic techniques to make a presumptive diagnosis. Typically, LSD laboratory testing involves virus isolation (VI), fluorescent antibody testing (FAT), electron microscopy, polymerase chain reaction (PCR), virus neutralization tests (VNT) and enzyme-linked immunosorbent assays (ELISA) [18]. Despite most of these tests being reliable and sensitive, they may not be easily accessible in some developing nations, though PCR has become cheaper and therefore more accessible. In addition, some of the serological tests have low specificity owing to cross-reactions between *Parapoxvirus* and *Capripoxvirus* [19]. Furthermore, these diagnostic tests require adequate financial, infrastructural, human resources and an adequate information system that are challenging to introduce under the current Ugandan setting. Therefore, control measures through vaccination and animal movement restrictions remain as the most practical options to control LSD in Uganda. However, LSD control through vaccination may be endangered by improper use of vaccines and by reports of partial protection of current LSD vaccines [20, 21]. Hence, the need for undertaking genetic characterization of LSDV during outbreaks to understand the genetic variation of field isolates. This genetic variation will give insights into the level of transboundary circulation of viruses, help identify disease hotspot areas and provide data which can be used to identify the origin of the LSDVs which caused outbreaks in Asia and South Eastern Europe.

Sensitive and specific molecular methods targeting p32, RPO30 and GPCR genes have been used to detect and characterise LSDV and other Capripoxviruses [22]. The G-protein-coupled chemokine receptor (GPCR) gene is one of the variable genes within Capripoxviruses [23] and is an appropriate target for genetic distinction between Capripoxviruses [24]. The suitability of the GPCR gene for host range phylogenetic grouping was described by Le Goff et al 2005 [25] and has been used by various authors to characterise Capripoxviruses [21, 22, 26–29]. The GPCR gene encodes a protein related to the G-protein-coupled chemokine receptor subfamily. The protein has the main structural features of the superfamily of G-protein-coupled chemokine receptors, such as seven hydrophobic areas and cysteine residues in the first and second extracellular loops. Even though previous studies have explored the epidemiology of LSDV in Uganda [30, 31], there is no data on the molecular characterization of circulating LSDV viruses. These data are important for understanding molecular epidemiology and vaccine design for disease control. In this study, we applied molecular methods to confirm LSDV infections from six outbreaks which occurred in different districts of Uganda 2017–2018 and performed phylogenetic analysis of LSDV GPCR gene, amplified from cattle samples obtained during these outbreaks.

## Results

### Field observations and confirmation of cases

**Fig. 1:**
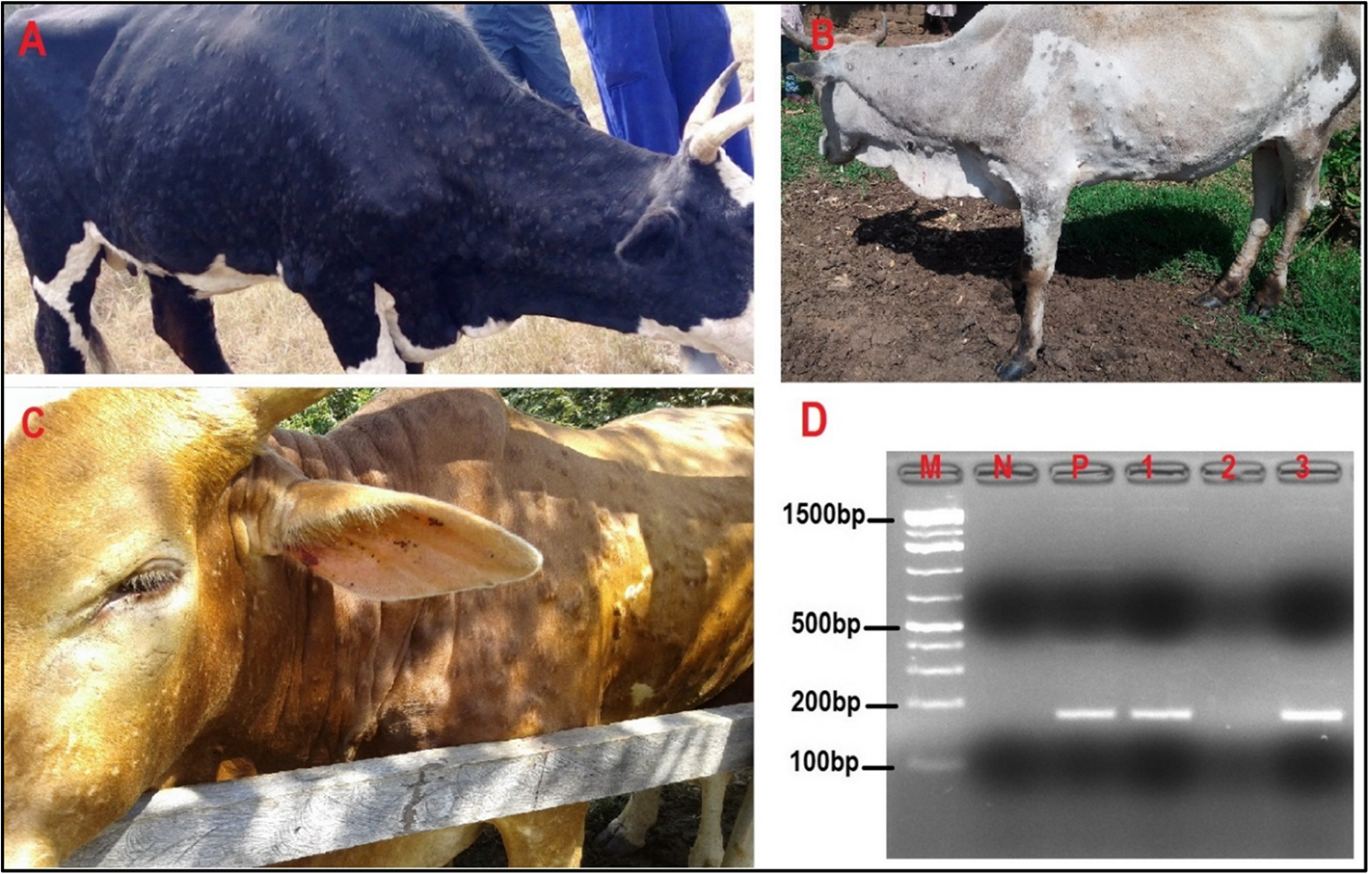
Lumpy skin disease virus, observed clinical signs and molecular (PCR) confirmation results: Cattle showing characteristic LSDV clinical signs; nodular skin lesions covering the whole body; and lacrimal discharge (panel A shows a cow with nodular skin lesions covering the whole body, panel B shows skin nodules on the neck and fore body and panel C shows skin nodules covering the whole body and lacrimal discharge). Panel D; PCR results showing a 192 bp fragment of the LSDV P32 gene, Lane M is a 100 bp molecular ladder (GeneDireX Inc., UK), lane N is a negative control, lane P a positive control. Lane 2 is a negative sample, while lanes 1 and 3 are samples positive for LSDV. All PCR products were run in 1.5% agarose gel

**Fig. 2:**
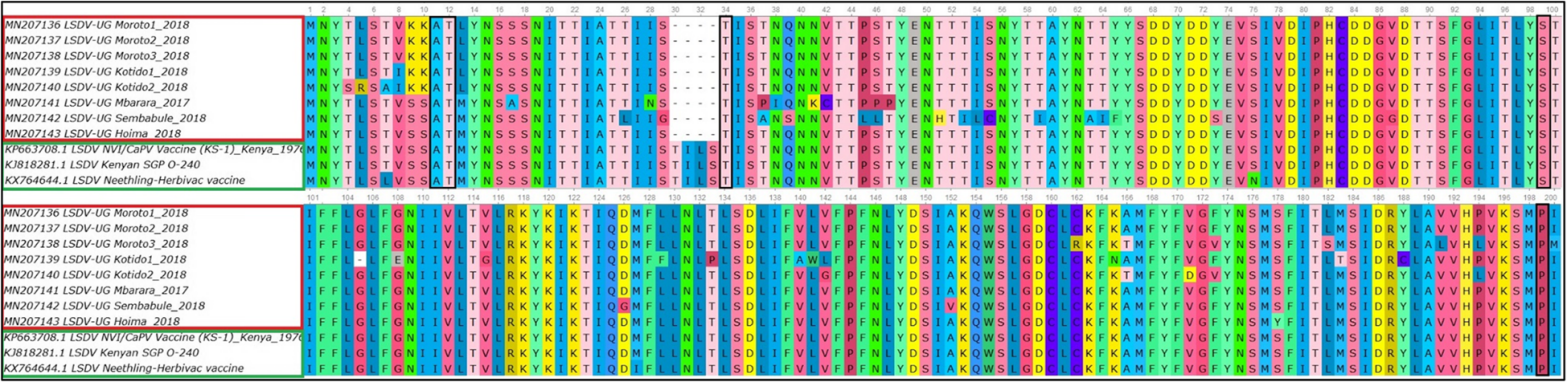
Multiple sequence alignment of GPCR sequences from Ugandan isolates and LSDV vaccine strains, showing positions of LSDV signature amino acid sequences A11, T12, T34, S99 and P199. Locations of the signature sequences are marked in a black horizontal rectanglar shape

Six suspected LSD outbreaks were investigated in five districts of Uganda in 2017 and 2018. A single outbreak was investigated in 2017 in Mbarara district, while in 2018, five outbreaks were investigated; in Hoima (one outbreak), Kotido (two outbreaks), and Moroto (two outbreaks). In Moroto district the two outbreaks investigated occurred in three herds. Of these three herds, two herds were from the same village (Matheniko-Rupa) and were therefore considered one outbreak (Additional file 1). The common clinical signs observed in cattle suspected to have LSDV were fever, depression, enlarged superficial lymph nodes, loss of appetite, circumscribed skin nodules on different parts of the body, lacrimation, nasal discharges and decrease in body weight (Fig. 1), (Table 1), (Additional file 1). The six (6) different outbreaks affected eight (8) cattle herds. In the affected herds, twenty three (23) suspected cases were sampled. Out of the 23 suspected cases, 15 were confirmed positive by PCR (65.2%). These confirmed cases were subjected to a second PCR targeting the GPCR gene (Additional file 2), sequenced and the sequences translated to amino acids to confirm presence of unique LSDV signature sequences (Fig. 2). One GPCR gene sequence from each of the eight outbreak herds was analysed in this study.

**Table 1:** Description of outbreaks, location of herd and descriptive data collected from the suspected cases of Lumpy skin disease investigated between 2017 and 2018

Date of Investigation	Location	Setting	Species/breed	Number of Cattle sampled	Clinical signs observed	Sample (s) collected
January 27, 2018	Hoima	Farm	Cattle, Ankole	2	pyrexia, nodular skin lesions	Skin Biopsy, blood
December 8, 2018	Kotido	Nomadic herd	Cattle, Zebu	2	pyrexia, nodular skin lesions, generalized enlarged lymph nodes	Skin scab, blood
December 8, 2018	Kotido	Nomadic herd	Cattle, Zebu	2	pyrexia, nodular skin lesions on the neck and shoulder	Skin Scab, blood
August 21, 2017	Mbarara	Farm	Cattle, Friesian	5	pyrexia, nodular skin lesions, ocular discharges	Skin Biopsy, blood
December 7, 2018	Moroto	Nomadic herd	Cattle, Zebu	4	pyrexia, nodular skin lesions	Skin Biopsy, blood
December 7, 2018	Moroto	Nomadic herd	Cattle, Zebu	3	pyrexia, nodular skin lesions	Skin Biopsy, blood
December 7, 2018	Moroto	Nomadic herd	Cattle, Zebu	3	pyrexia, nodular skin lesions	Skin Biopsy, blood
March 4, 2018	Sembabule	Farm	Cattle, Friesian cross	2	pyrexia, nodular skin lesions	EDTA blood

### Phylogenetic analysis of LSDV using the GPCR gene

**Fig. 3:**
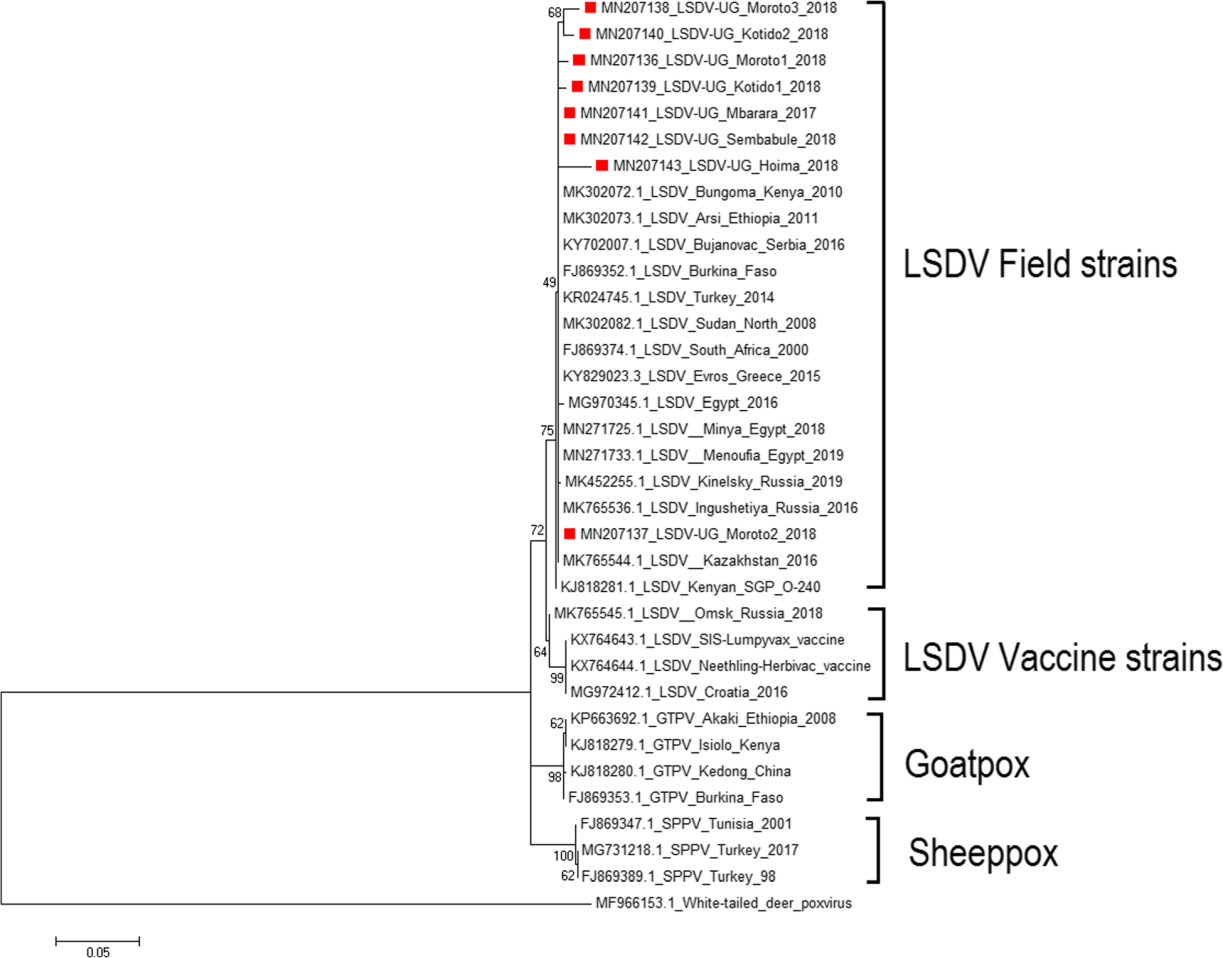
Phylogenetic tree showing the relationship between LSDV GPCR gene sequences from Uganda, marked with red square, with other *Capripoxvirus* GPCR gene sequences from GenBank. A homologous gene sequence from Deerpox virus retrieved from GenBank was used as out-group to root the tree

Phylogenetic analyses were performed to determine the phenetic relationship among Ugandan isolates and other Capripoxviruses whose sequences were obtained from GenBank (Table 2). Phylogenetic analysis clustered LSDV outbreak and vaccine strains into separate clades within the *Capripoxvirus* family. Sheeppox virus and goatpox viruses also clustered in separate clades (Fig. 3). The field LSDV isolates from Ugandan cattle were more closely related to other LSDV sequences from Africa (Kenya, Ethiopia, Egypt and Sudan) and from Europe (Greece and Russia). The Ugandan outbreak samples showed nucleotide sequence identities between 94.35 and 99.01% when compared to outbreak sequences from GenBank. When compared to vaccine strain sequences, nucleotide identities were 2–3% lower than when compared to outbreak sequences, and gave identities between 91.64 and 96%. The diversity of sequences from Uganda is higher when compared amongst each other and did not all cluster with each other (Fig. 3).

**Table 2:** Details of selected Capripoxvirus sequences used for phylogenetic analysis based on G-protein-coupled chemokine receptor gene

Isolate name	Sequence Length (bp)	Country of origin	Year of collection	Host species	GenBank accession no.
LSDV	1134	Russia	2015	Bovine	MH893760.2
LSDV	1134	Kenya	2010	Bovine	MK302072.1
LSDV	1134	Ethiopia	2011	Bovine	MK302073.1
LSDV	1134	Serbia	2016	Bovine	KY702007.1
LSDV	1134	Burkina Faso	2010	Bovine	FJ869352.1
LSDV	1038	Turkey	2014	Bovine	KR024745.1
LSDV	1134	Kenya	2014	Bovine	KJ818281.1
LSDV	1134	Sudan	2008	Bovine	MK302082.1
LSDV	1134	South Africa	2010	Bovine	FJ869374.1
LSDV	1134	Greece	2015	Bovine	KY829023.3
LSDV	1134	Egypt	2016	Bovine	MG970345.1
LSDV	507	Egypt	2018	Bovine	MN271725.1
LSDV	507	Egypt	2019	Bovine	MN271733.1
LSDV	1134	Russia	2019	Bovine	MK452255.1
LSDV	791	Russia	2016	Bovine	MK765545.1
LSDV	779	Kazakhstan	2016	Bovine	MK765544.1
LSDV vaccine	1146	South Africa	2016	Bovine	KX764644.1
LSDV vaccine	1146	South Africa	2016	Bovine	KX764643.1
LSDV vaccine	1146	Croatia	2016	Bovine	MG972412.1
SPPV	1056	Turkey	2017	Sheep	MG731218.1
SPPV	1125	Turkey	1998	Sheep	FJ869389.1
SPPV	1125	Tunisia	2001	Sheep	FJ869347.1
GTPV	1146	Ethiopia	2008	Goat	KP663692.1
GTPV	1146	Kenya	2014	Goat	KJ818279.1
GTPV	1146	China	2014	Goat	KJ818280.1
GTPV	1146	Burkina Faso	2010	Goat	FJ869353.1
Deerpox	723	USA	2018	White-tailed deer	MF966153.1

### Comparison of outbreak samples with vaccine strains

**Fig. 4:**
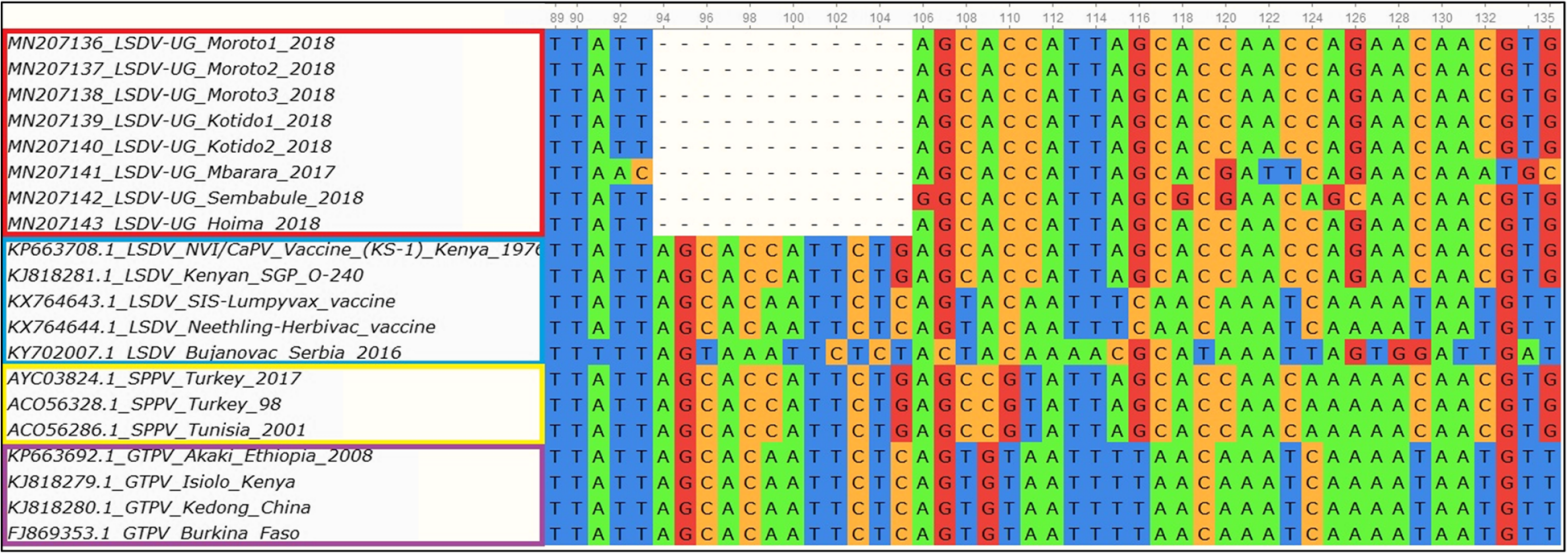
Multiple sequence alignment of GPCR gene sequences of Ugandan LSDV field isolates, vaccine strains, Sheeppox and Goatpox virus. A 12 bp nucleotide (position 94 to 105) deletion unique to only LSDV from this study is shown. Sequences from Uganda are marked with a red rectangle, vaccine strains in blue, Sheeppox in yellow and Goatpox in purple

The analysis of the GPCR gene showed major sequence differences between the vaccine strain and the field isolates. A 12 bp nucleotide deletion (Fig. 4) was found in the GPCR gene for all outbreak isolates collected from cattle in Uganda while no such deletion was observed in the vaccine strains.

## Discussion

This study presents the first results of molecular detection and phylogenetic analysis of lumpy skin disease virus from outbreaks in Uganda, which occurred between 2017 and 2018. In Uganda, LSDV is endemic and currently control of the disease is through quarantine restrictions and vaccination [7]. Vaccination is not done by the government which affects the coverage and quality of vaccines used. LSD vaccination is conducted by those farmers that can access and/or afford the cost of vaccines and delivery of vaccines into the animals. This limitation poses a danger of misuse or abuse of the use of vaccines which may result in reversion of vaccine strains into virulent strains, consequently being responsible for new outbreaks [17]. This study therefore presents insights into the current viruses responsible for outbreaks in the country and compares them to viruses from neighboring countries and with LSDV vaccine strains.

During the study, a conventional PCR using primers that target a 192 bp region of the LSDV P32 gene was used to confirm presence of LSDV viral DNA from suspected clinical cases presenting with multiple skin nodules. Not all the samples obtained from suspected clinical cases tested positive by PCR, Fifteen (15) samples out of twenty three (23) tested positive. This is likely because only a blood sample was taken from some animals, and these blood samples tested negative. The reason for a negative result from a blood sample taken from a clinically sick animal could be that the virus is known to be present in blood for a short time 4–11 days, hence may have been missed [32]. This result is however in agreement with previous reports by Zeynalova et al 2016 [33], who concluded that skin nodules are better samples for PCR detection of LSDV than blood samples.

The sequences obtained from the GPCR gene amplicons were translated into corresponding amino acid sequences and when checked for presence of unique signatures associated with LSDV as reported by Le Geoff et al 2009 [34], these translated GPCR amino acid sequences showed these unique LSDV signatures (A11, T12, T34, S99, and P199) therefore further confirming LSDV (Fig. 2). To the best of our knowledge this is the first published study reporting LSDV in Uganda using molecular methods. BLAST analysis revealed high sequence homology 94.35–99.01% between Ugandan LSDV sequences and sequences in GenBank. Phylogenetic analysis of GPCR gene sequences was able to group the Capripoxviruses into three distinct groups (LSDV, SPPV and GTPV). Phylogenetic analysis further showed that LSDVs from outbreaks in Uganda grouped with LSDV isolates from Kenya, Egypt, Sudan, Ethiopia, Turkey, Serbia, Russia, Kazakhstan and Greece (Fig. 3). These sequences were however most closely related to sequences from Kenya and Sudan when compared by nucleotide identity, therefore suggesting that the same LSDVs are responsible for outbreaks across borders. This is highly likely because of the porous nature of the border between Kenya and Uganda. In the northeastern border of Uganda and Kenya, there are pastoral communities who move across the borders in search of pasture and water for their cattle therefore easily spreading diseases such as LSD. It is interesting to note that the diversity of the GPCR sequences from this study is higher than what has been previously observed, where most of the GPCR sequences in GenBank are almost identical. We however did not observe any trend in virus circulation amongst the different livestock production systems in Uganda. This is more likely due to the small number of LSDV sequences being compared in this study and comparison of only a single gene.

Comparison of GPCR gene sequences from this study with GPCR sequences from LSDV vaccine strains (obtained from GenBank) commonly used in the East African region revealed a 12 bp deletion between nucleotide position 94 and 105 in the outbreak sequences when compared to the LSDV vaccine strains. This finding is similar to reports by Gelaye et al 2015 [21] who reported similar deletions in the GPCR gene of virulent LSDVs. The inferences for Uganda are that the current LSDVs causing outbreaks are genetically different from viruses in the Neethling vaccines used in the country. Nonetheless, in order to confirm these strain variations between the vaccine and the wild type virus, further diagnostic testing, along with the sequencing of several LSDV genes, must be done, as this study compared only a single gene. Our findings further indicate that despite the weak regulations governing vaccine acquisition in Uganda, the current outbreaks are most probably caused by wild type virus which differ genetically from vaccine strains. This also means that a differential diagnostic method can be developed based on this sequence difference between vaccine and wild type virus, and this can be used as a tool to monitor vaccination [35]. Vaccination is reported as the best way to control LSDV and it can be done with attenuated LSDV, sheeppox and goatpox viruses [36, 37]. It is however still necessary to evaluate the efficacy of the currently approved LSDV vaccines under field conditions in Uganda, before mass vaccination can be rolled out.

## Conclusions

This is the first study on molecular detection and phylogenetic analysis of LSDV in Uganda, using the GPCR gene. These findings hint at genetically similar LSDV viruses circulating in the East African region, and this emphasizes the transboundary nature of LSDV. In addition, we note here that based on a single gene comparison, outbreak viruses differ from vaccine strain viruses. In order to fully understand the molecular epidemiology of LSDV in Uganda, further characterization is required using whole genome sequencing.

## Methods

### Study area, origin of samples and sample collection

**Fig. 5:**
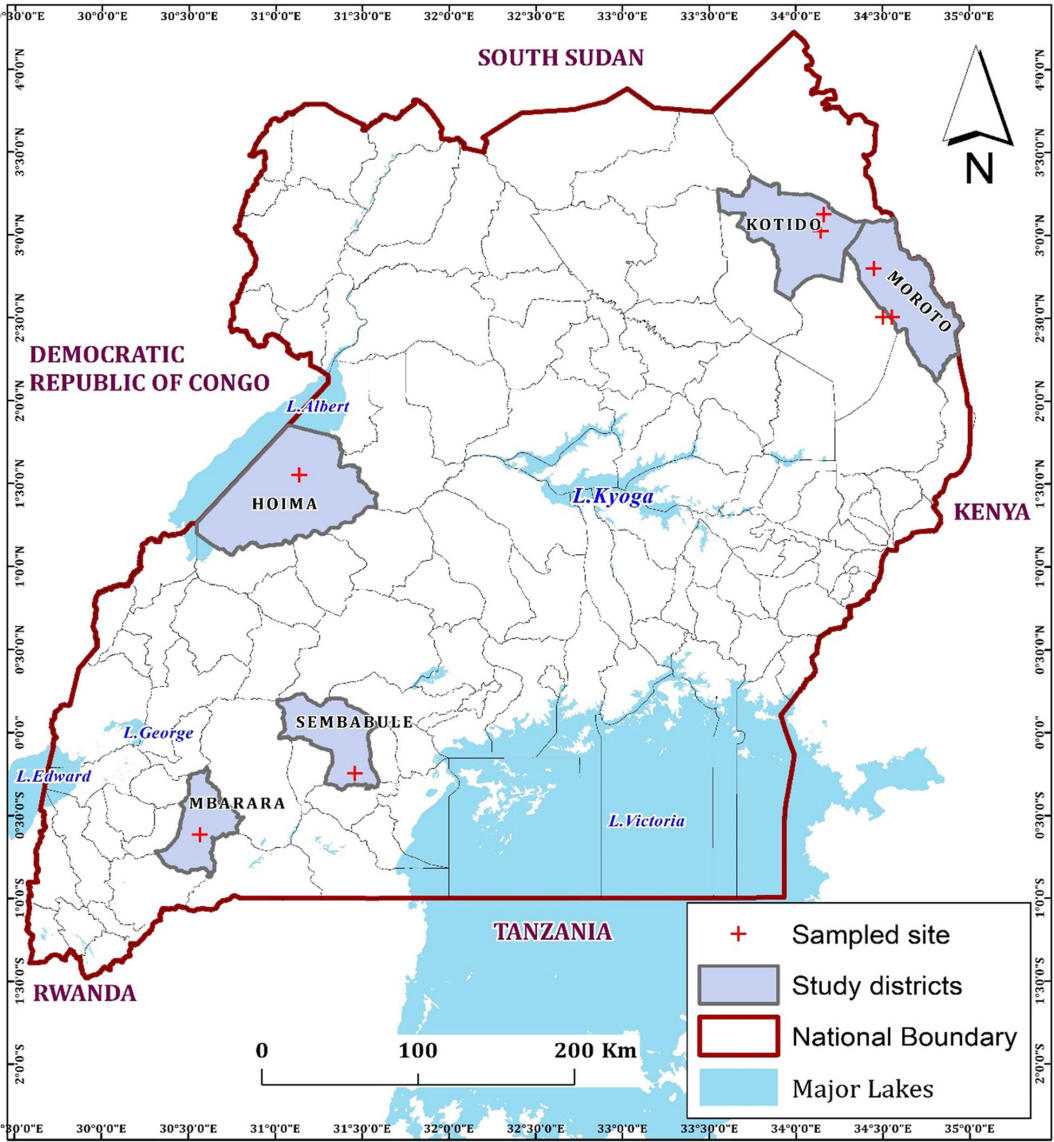
Location of study area. Districts where outbreaks occurred are shown in grey with a bold dark boundary, coordinates of sampled sites are marked in a red cross. (The image depicted in Figure 5 is our own)

The study was undertaken in five districts of Moroto, Kotido, Mbarara, Sembabule and Hoima (Fig. 5). The study districts were located in North East (Moroto, Kotido), Central (Sembabule) and Western (Mbarara, Hoima) regions. Uganda is divided into 121 districts found in four major administrative regions: North, East, Central and West. Each region is primarily characterized by different livestock production systems: Northern region is characterised by agro-pastoral and pastoral system; Eastern region is mainly agro-pastoral; Central and west by agro-pastoral, semi-intensive and ranching [38]. We sampled twenty-three cattle suspected to be affected by LSD from six field outbreaks in the 5 districts during the period from August 21, 2017 to December 8, 2018. The sampled animals were not previously vaccinated against LSDV. Samples of skin biopsies and scabs were collected in sterile cryovials containing 1 ml Minimum Essential Medium (MEM), Merck-Sigma, USA and whole blood in EDTA tubes. These samples were collected aseptically as described by the OIE [39]. In addition, information on clinical signs of the suspected LSD affected animals was recorded. Each sample was given a unique sample ID, placed in a cooler box with ice and transferred to the molecular biology laboratory, College of Veterinary Medicine Animal Resources and Biosecurity (COVAB) and stored at − 80 °C for further molecular analysis.

### DNA extraction and PCR confirmation of LSDV

The samples (skin biopsies, scabs and whole blood) were thawed at room temperature. Skin biopsy and scab samples were cut with a sterile scalpel blade into small pieces weighing about 400 mg and homogenized in 500 μl of sterile 1X PBS solution, pH 7.4. Total DNA was extracted from tissue homogenates and 200 μl blood aliquots using a DNeasy Blood and tissue kit (Qiagen, Germany) following manufacturer’s instructions. PCR was then performed to confirm presence of LSDV specific nucleic acid by amplifying a 192 bp region in the p32 gene using a pair of primers; forward primer, 5′-TTTCCTGATTTTTCTTACTAT-3′ and reverse primer, 5′-AAATTATATACG TAAATAAC-3′, and PCR conditions as described by Ireland and Binepal (1998) [40]. The PCR reaction was set up in a 50 μl final volume containing 25 μl of 2X MyTaq™ Red mix (Bioline, United Kingdom), 1.5 μl of each 10 μM primer concentration, 19.5 μl of PCR water, and 2.5 μl of extracted DNA. The PCR was performed in a Bio-Rad S1000 ThermoCycler (Bio-Rad, United Kingdom). The PCR conditions had an initial denaturation step of 94 °C for 5 min, followed by 34 cycles of denaturation at 94 °C for 1 min, annealing at 50 °C for 30 s, extension at 72 °C for 1 min and a final extension step of 72 °C for 5 min. The PCR products were viewed on a 1.5% Agarose gel to confirm LSDV positive samples, with a band size of 192 bp.

### PCR amplification of the GPCR gene

A second PCR was carried out on all positive samples to amplify the GPCR gene for phylogenetic analysis. This was done using primers designed by Le Goff et al., 2009 [24], with the following sequences (5′- TTAAGTAAAGCATAACTCCAACAAAAATG-3′ and 5′-TTTTTTTATTTTTTATCCAATGCTAATACT-3′), that were designed to amplify a fragment between nucleotide 6961–8119 in the LSDV genome [23]. An additional primer pair (5′-GATGAGTATTGATAGATACCTAGCTGTAGTT-3′ and 5′-TGAGACAATCCA AACCACCAT-3′) was positioned internally for sequencing [24]. The DNA amplification of the GPCR gene was performed in a 50 μl volume in the presence of 25 μl of 2X MyTaq™ Red mix (Bioline, UK), 1.5 μl of each 10 μM primer concentration, 19.5 μl of nuclease free water, and 2.5 μl of DNA extract. The PCR amplification of the GPCR gene involved an initial denaturation at 96 °C for 5 min followed by 35 cycles of final denaturation at 95 °C for 30s, annealing at 50 °C for the 30s, and extension at 72 °C for 30s as previously described. All PCR products were resolved on 1.5% agarose gel against HyperLadder™ 100 bp DNA ladder (Bioline, United Kingdom) at 125 V in 1X Tris-Acetic acid-EDTA (TAE) buffer containing 0.5 μg/ml ethidium bromide for 35 min. The gels were visualized using the ENDURO™ gel documentation system (LaboNet, USA).

### Nucleotide sequencing and analysis

Following agarose gel electrophoresis on a 1.5% agarose gel, amplification products of the expected size were identified against a molecular weight marker. DNA bands of correct size were excised and purified by gel purification (Qiagen, Germany), as specified by the manufacturer, and sent to Inqaba Biotec (South Africa) for Sanger sequencing. The sequences obtained were checked for quality and the ends of the sequences trimmed using BioEdit software (Ibis Biosciences, Carlsbad, CA, USA). The trimmed sequences were then checked for similarity with other LSDV GPCR sequences in GenBank using the National Center for Biotechnological Information’s (NCBI) web-based Basic Local Alignment Search Tool (BLASTn). These nucleotide sequences were then further checked for LSDV-specific signatures by translating them to amino acid sequences followed by multiple sequence alignment using MUSCLE found at the EMBL-EBI web server. Phylogenetic analysis was done using Molecular Evolutionary Genetics Analysis (MEGA) version 6 (Pennsylvania, USA). Thirty four (34) Capripoxvirus and one Deerpox GPCR sequence (used to root tree) were selected from GenBank to be used for phylogenetic analysis. After BLAST, LSDV sequences were selected based on nucleotide similarity and origin of isolates, so as to have representative sequences from East Africa, the rest of Africa and Eurasia. We also selected sequences from LSDV vaccine strains, goatpox and sheeppox virus. A phylogenetic tree was constructed using the maximum likelihood method based on Tamura 3 parameter model, with 1000 bootstrap replications. The tree was drawn to scale, with branch lengths in the same units as those of the evolutionary distances used to infer the phylogenetic trees. All sequences were submitted to GenBank and can be found under the accession numbers MN207136-MN207143.

## Supplementary information

**Additional file 1.** Table showing details of all samples taken from cattle showing clinical signs consistent with Lumpy skin disease

**Additional file 2.** PCR amplification of the LSDV GPCR gene. PCR results showing a 1150 bp fragment of the LSDV GPCR gene. Lane M is a 50 bp molecular ladder (Hyper Ladder, Bioline UK), Lane P is positive control (LSDV vaccine), Lane N is negative control, lanes 1–5 are positive samples. PCR products were run in 1.5% agarose gel

## Data Availability

The datasets generated and/or analysed during the current study are available in the National Center for Biotechnology Information (NCBI) repository, under these GenBank accession numbers MN207136, MN207137, MN207138, MN207139, MN207140, MN207141, MN207142, and MN207143.

## Abbreviations

COVAB: College of Veterinary Medicine Animal Resources and Biosecurity
LSD: Lumpy Skin Disease
LSDV: Lumpy Skin Disease Virus
MAAIF: Ministry of Agriculture Animal Industry and Fisheries
