# Supplementary material for: Molecular detection and phylogenetic analysis of lumpy skin disease virus from outbreaks in Uganda 2017–2018
Source: BMC Vet Res. 2020 Feb 21;16:66. doi: 10.1186/s12917-020-02288-5 (PMC7035724; doi:10.1186/s12917-020-02288-5)
Supplement: Supplementary file 1 — Additional file 1. Table showing details of all samples taken from cattle showing clinical signs consistent with Lumpy skin disease [file 12917_2020_2288_MOESM1_ESM.docx]

| District | Outbreaks Investigated | Subcounty/Village | Cattle with clinical signs | Cattle sampled | Herd size | Samples taken |
| --- | --- | --- | --- | --- | --- | --- |
| Hoima | 1 | Kabale/Nyamasoga | 4 | 2 | 109 | Blood, skin biopsies |
| Kotido | 1 | Rengen/Lokudeli | 6 | 2 | 35 | Blood, scabs |
|  |  | Rengen/Nabwalin | 2 | 2 | 40 | Blood, skin biopsies |
| Mbarara | 1 | Kashari | 12 | 5 | 57 | Blood, skin biopsies |
| Moroto | 2 | Matheniko/Nadunget | 7 | 4 | 78 | Blood, scabs, skin biopsies |
|  |  | Matheniko-Rupa | 3 | 3 | 80 | Blood, scabs, skin biopsies |
|  |  | Matheniko | 8 | 3 | 43 | Blood, scabs, skin biopsies |
| Sembabule | 1 | Malere/kyabajanga | 2 | 2 | 40 | Blood |
