# Supplementary figures and images for: Molecular detection and phylogenetic analysis of lumpy skin disease virus from outbreaks in Uganda 2017–2018
Source: BMC Vet Res. 2020 Feb 21;16:66. doi: 10.1186/s12917-020-02288-5 (PMC7035724; doi:10.1186/s12917-020-02288-5)

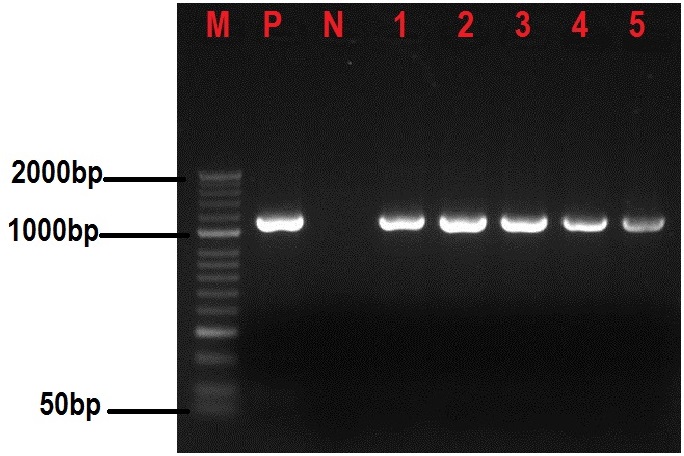

Supplement: Supplementary file 2 — Additional file 2. PCR amplification of the LSDV GPCR gene. PCR results showing a 1150 bp fragment of the LSDV GPCR gene. Lane M is a 50 bp molecular ladder (Hyper Ladder, Bioline UK), Lane P is positive control (LSDV vaccine), Lane N is negative control, lanes 1–5 are positive samples. PCR products were run in 1.5% agarose gel [file 12917_2020_2288_MOESM2_ESM.docx]
